# Measles-Rubella Supplementary Immunization Activity Readiness Assessment — India, 2017–2018

**DOI:** 10.15585/mmwr.mm6726a3

**Published:** 2018-07-06

**Authors:** Vandana Gurnani, Pradeep Haldar, Sudhir Khanal, Pankaj Bhatnagar, Balwinder Singh, Danish Ahmed, Mohammad Samiuddin, Arun Kumar, Yashika Negi, Satish Gupta, Pauline Harvey, Sunil Bahl, Alya Dabbagh, James P. Alexander, James L. Goodson

**Affiliations:** ^1^Ministry of Health and Family Welfare, Government of India, New Delhi, India; ^2^Immunization and Vaccine Development, World Health Organization Regional Office for South-East Asia, New Delhi, India; ^3^National Polio Surveillance Project, India Country Office, World Health Organization, New Delhi, India; ^4^India Country Office, United Nations Children’s Fund, New Delhi, India; ^5^Immunization and Vaccine Development, World Health Organization, Geneva, Switzerland; ^6^Global Immunization Division, Center for Global Health, CDC.

In 2013, during the 66th session of the Regional Committee of the World Health Organization (WHO) South-East Asia Region (SEAR), the 11 SEAR countries* adopted goals to eliminate measles and control rubella and congenital rubella syndrome by 2020^†^ (*1*). To accelerate progress in India (*2*,*3*), a phased^§^ nationwide supplementary immunization activity (SIA)^¶^ using measles-rubella vaccine and targeting approximately 410 million children aged 9 months–14 years commenced in 2017 and will be completed by first quarter of 2019. To ensure a high-quality SIA, planning and preparation were monitored using a readiness assessment tool adapted from the WHO global field guide** (*4*) by the India Ministry of Health and Family Welfare. This report describes the results and experience gained from conducting SIA readiness assessments in 24 districts of three Indian states (Andhra Pradesh, Kerala, and Telangana) during the second phase of the SIA. In each selected area, assessments were conducted 4–6 weeks and 1–2 weeks before the scheduled SIA. At the first assessment, none of the states and districts were on track with preparations for the SIA. However, at the second assessment, two (67%) states and 21 (88%) districts were on track. The SIA readiness assessment identified several preparedness gaps; early assessment results were immediately communicated to authorities and led to necessary corrective actions to ensure high-quality SIA implementation.

## Supplemental Immunization Activity Readiness Assessment Process

SIA readiness assessments were conducted in 24 (41%) of the 58 districts in the states of Andhra Pradesh (seven districts), Kerala (five), and Telangana (12). In addition, 74 (72%) of 103 blocks^††^ in Telangana were selected for readiness assessments. Districts and blocks were selected for assessment based on low routine vaccination coverage, difficult-to-reach populations, high proportion of urban to rural population, and categorization as polio high-risk based on polio risk assessments.

The assessments were conducted by teams coordinated by the WHO India Country Office. The teams included members from the India Ministry of Health and Family Welfare, especially the Immunization Technical Support Unit, National Institute of Health and Family Welfare, and senior immunization program officers from other states; United Nations agencies, including WHO, United Nations Children's Fund (UNICEF), and United Nations Development Program; and nongovernmental organizations, including John Snow Inc., Global Health Strategies, CORE Group Polio Project, and others.

The India SIA readiness assessment tool and checklists were adapted from the WHO field guide for planning and implementing SIAs (*4*) according to the India national measles-rubella SIA operational guidelines, for use at the national, state, district, and block levels. Assessment teams reviewed preparations in planning and coordination, advocacy, accountability, management of adverse events following immunization, vaccines and logistics management, funding, and communication, using checklists modified at each level based on expected functions of SIA components for that level (Table 1). The checklists included questions with possible answers of “yes” or “no.” The overall percentage of affirmative responses was calculated, and the assessed area was categorized as “on track” (≥80%), “needs work” (60%–79%), or “not ready” (<60%).

**TABLE 1:** Questions on supplementary immunization activities readiness assessment checklist, by component — India, 2017–2018

Component	Activity
**Planning and coordination**	State/District SIA Steering Committee met at least once?
	Did all essential government officials participate in at least one State Task Force for Immunization (STFI) meeting?*
	Circle those who did not participate: Permanent Secretary/State Education Officer/State Program Officer/Women and Child Development/Integrated Child Development Services/Minority Welfare Officer*
	Did essential non-governmental stakeholders participate in at least one STFI meeting?*
	Circle those who did not participate: Indian Medical Association (IMA)/Indian Academy of Pediatrics (IAP)/private practitioners/LIONS International/religious leaders.*
	State/district Immunization Officer or other state level monitors using state checklist-A for tracking progress of state level preparedness?
	State/district Immunization Officer using checklist-B for tracking progress by visiting the priority districts?*
	State/district monitors identified for visiting the priority districts for assessing the SIA preparedness?
	State/district Education Officer communicated with all District Education Officer?
	State/district Program Officer communicated with all Child Development Project Officers?
	Has the state committee for adverse events following immunization (AEFI) met at least once?
**Sensitization meetings**	Sensitization meeting held with heads of IMA and IAP, including leading private practitioners?*
	Sensitization meeting held with district level Education Officers?
	Coordination meeting with state level representatives of public schools, private schools’ associations, religious institutions, etc.?*
**Vaccine logistics and management**	Adequate quantity of vaccine and diluents available per microplan? (consider planned staggered distribution of vaccine)
	Adequate quantity of auto-disable syringes and mixing syringes available per microplan? (consider planned staggered distribution of vaccine)
	Adequate quantity of indelible marker pens available per microplan? Vaccine distribution plan available for districts?
**Funds**	Has state received funds from the national level?
	Has state disseminated financial guidelines to all districts?
**Communication planning**	Is there a nodal officer, other than State EPI Officer, designated for SIA communication planning at state level?
	At least one joint meeting held for secretaries of Health, Education, other department? (check for official circular)
	State communication core group formed and held at least one meeting? (verify meeting minutes)*
	SIA communication plan prepared in a template as per operational guidelines?
	All districts/blocks have submitted communication plan in prescribed template?
	Received guidelines for communication activities including financial for SIA and shared with all districts? (check for official circular)*
	State/district implementing communication plan for underserved communities? (identified influencers, religious and educational institutions for support)*
	Was there discussion on communication planning in STFI? (verify meeting minutes)
**Communication and social mobilization**	Printed and distributed all IEC (Information, Education, and Communication) materials or guidelines?
	Identified local celebrities or champion for SIA? (verify how involved in SIA)
	State/district launch or inauguration for SIA? (confirm date for launch)
**Advocacy**	Sensitization meeting with religious leaders or influencers planned/held?
**Media and social media**	State/district has identified media spokesperson for the SIA?
	Media workshop planned at state level for SIA? (confirm dates for media workshop)
	Is an official or agency regularly tracking media and social media for SIA and immunization messages? (collect related news articles)*
	Task force for social media was formed? (confirm at least one responsible person designated at state level for managing social media)
	WhatsApp group(s) was formed for health, education, and immunization-related sectors?
	Facebook page was created for the SIA? (check the page for SIA post)*

* These variables were considered to be critical and were evaluated subjectively by the assessment team to decide "go" or "delayed go" for an area marked as "needs work." The checklists used at state, district, planning unit, and school levels were modified to reflect the level-specific role and function for each component.

The first readiness assessment was conducted 4–6 weeks before the SIA and the second, 1–2 weeks before the SIA. A decision either to start the SIA on the designated date or to delay the SIA until preparations were complete was made at the district and state levels, based on the second assessment score and categorization of the district or state assessed. Those areas categorized as on track were permitted to start the SIA (“go”); those categorized as not ready were delayed (“delayed go”); and those categorized as needing work either started or delayed the SIA, based on subjective evaluation by the assessment team of critical gaps and level of commitment to taking corrective actions in a timely manner. At the end of the assessment, evidence-based feedback from the teams was shared with health and administration leaders at district, state, and national levels to facilitate decision-making for strengthening the quality of this and future SIAs.

## Supplemental Immunization Activity Readiness Assessment Results

At the first assessment, none of the three states and none of the 24 districts was on track (Table 2). The challenges most frequently identified during the preparedness assessment were lack of logistics and training materials and nonengagement of schools. Based on feedback provided, state-level program managers initiated corrective actions in all districts. At the second assessment, Kerala and Telangana states were on track; Andhra Pradesh needed work and had to delay the start of the SIA to provide an additional week for preparation. Overall, 19 (79%) of the 24 districts were on track (including information, education, and communication [IEC] readiness), four (17%) needed additional work and undertook minor corrective actions, and one (4%) was not ready and had a delayed go.

**TABLE 2:** Supplementary immunization activity readiness assessment* results — three states, India, 2017–2018

SIA readiness assessment results	State (no. of districts)		
	Andhra Pradesh (7)	Kerala (5)	Telangana (12)
**First assessment**			
**Not ready, no. (%)**	5 (71)	1 (20)	10 (83)
**Needs work, no. (%)**	2 (29)	4 (80)	2 (17)
**On track, no. (%)**	0 (0)	0 (0)	0 (0)
**Key findings**	State level trainings not started	No SIA logistics plan available	IEC materials not available
	IEC materials not available	No schools aware of SIA	No clarity on SIA financial guidelines
	Most schools not informed	Trainings conducted without training materials	Private schools not on board
	Medical fraternity not involved and informed about SIA	High level of vaccine hesitancy and frank refusal in one district	Informal educational institutions, religious schools, madrasas not in target population
	Low level SIA awareness	No clarity on financial guidelines for local implementers	Low level preparedness for management of AEFI
			Language barriers
			Lack of SIA awareness
			Vaccine hesitancy in minority communities
**Actions taken**	Video conference with all districts by the principal secretary and by each district to all blocks to discuss assessment findings and plan corrective actions	SIA logistics made immediately available to the districts	Video conference with all deputy commissioners, chief medical officers, and district immunization officers requesting immediate corrective actions
	Principal secretary visited all high-risk districts to get firsthand information on preparedness progress and next steps	Microplans reviewed in all areas; additional field monitors deployed in high-risk districts and blocks	Meeting with district education officers to develop plan; directives for noncompliant schools, meeting with heads of madrasas organized to encourage SIA participation
	Operational communication plan developed with all partners; all district microplans reviewed	Additional communication and social mobilization officers mobilized in areas with vaccine hesitancy and refusal	Prominent talk show personalities appear on local television channels; media release in Urdu language; video of prominent opinion leaders and religious leaders developed and circulated through social media platform
	Medical and Indian Academy of Pediatrics invited to participate in process and promote SIA in local newspaper	Medical colleges and medical fraternity brought on board as support group to the SIA	District magistrates briefed on assessment results; called all immunization offices and received regular updates on progress to accelerate preparedness
			Senior state officers visited high-risk areas to accelerate preparedness activities
			District AEFI committee reactivated and capacity building done
			Administrative processes to print and deploy materials were fast-tracked. Orientation on financial guidelines
			Meeting with district governors of Lions Clubs International and request to adopt problematic schools to accelerate SIA preparedness and awareness
**Second assessment**			
**Not ready, no. (%)**	1 (14)	0 (0)	0 (0)
**Needs work, no. (%)**	3 (43)	0 (0)	1 (8)
**On track, no. (%)**	3 (43)	5 (100)	11 (92)
**Decision**	Delay	Move forward	Move forward
**% Administrative coverage, state (districts range)**	97 (86 to >100)	89 (87 to 98)	>100 (87 to >100)

**Abbreviations:** AEFI = adverse events following immunization; IEC = information, education, and communication; SIA = supplementary immunization activity.

* SIA readiness assessments during planning for phase 2 of the nationwide SIA using measles-rubella vaccine for children aged 9 months–14 years that started in 2017. The first readiness assessment was conducted at 4–6 weeks before the SIA and the second assessment at 1–2 weeks before the SIA. Checklists had questions with possible answers of “Yes” or “No.” Scoring was based on percentage of “Yes” responses, categorized as on track (≥80%), needs work (60%–79%), and not ready (<60%). Administrative coverage >100% indicated the intervention reached more persons than were in the estimated target population.

During the SIA, rapid convenience monitoring, a programmatic tool that identifies children not vaccinated during the campaign and compiles reasons for nonvaccination, determined that 9,912 (6.9%) of all 143,894 targeted children were not vaccinated during the SIA, including 7% (3,314 of 44,906) in Andhra Pradesh, 10% (1,943 of 19,408) in Kerala, and 6% (4,659 of 79,580) in Telangana (Figure). Among all unvaccinated children located through rapid convenience monitoring, the most frequently reported reason given by caregivers for not vaccinating was that the child was sick (3,715; 37%), followed by lack of awareness of the campaign (1,566; 16%). In Kerala, refusal accounted for approximately a quarter of children who were not vaccinated. The least frequently reported reason (209; 2%) for nonvaccination was SIA operational gaps (e.g., nonfunctioning vaccination sites, absent or late vaccinators, vaccine stock-outs, and other logistics issues) (Figure). Reported SIA administrative coverage was ≥95% in two states and 17 districts (Table 2).

**FIGURE:**
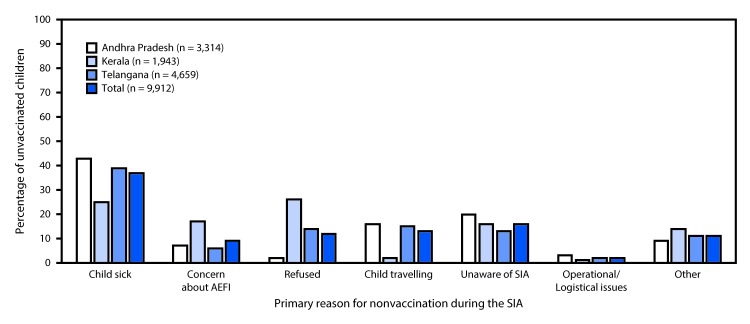
Percentage of unvaccinated children, by reported primary reason for nonvaccination* during supplementary immunization activity**^†^** (phase 2)**^§^** — Andhra Pradesh, Kerala, and Telangana states, India, 2017–2018 **Abbreviations:** AEFI = Adverse events following immunization; MR = measles-rubella; SIA = supplementary immunization activity. * Intra-SIA monitoring using rapid convenience monitoring. † Nationwide SIA using MR vaccine for children aged 9 months–14 years. ^§^ Phase 2 of phased nationwide SIA started in 2017 and to be completed by first quarter of 2019. Children targeted for vaccination during phase 2 of the SIA but not vaccinated included 7% in Andhra Pradesh, 10% in Kerala, and 6% in Telangana.

## Discussion

Experience with the SIA assessment in India demonstrated that the WHO SIA readiness assessment tool and procedures were useful for ensuring preparedness for implementation of a high-quality SIA. Corrective actions implemented after the first assessment, which found that two thirds of districts were not ready for the SIA, resulted in 79% of districts being on track by the second assessment. Providing feedback to key decision-makers immediately after the assessments helped with planning and allocation of resources and facilitated implementation of timely corrections. These midcourse corrections also might have resulted in further-reaching effects across each of the three states because of the statewide directives issued by immunization program managers for corrective actions in all districts to better prepare for this SIA and future SIAs.

As suggested in the global guidelines, decision-makers in India used the terminology “delayed go” rather than “no go” in states and districts assessed as not ready for the measles-rubella SIA, to provide positive reinforcement to immunization program personnel who needed additional time for preparation. Intra-SIA rapid convenience monitoring found that SIA operational gaps were the least common reason for children not being vaccinated, an indication of good preparation and implementation of campaign activities. The primary reasons for children not being vaccinated during the SIA were related to IEC gaps and challenges in addressing parental misperceptions and their lack of awareness of and availability for the SIA. These findings suggest that the WHO SIA readiness checklists section on IEC and communication strategies might need to be revised and expanded.

Although WHO global guidance recommends four to six assessments before an SIA to ensure readiness, in this setting, only two pre-SIA assessments were designed and conducted in each area. Because the SIA readiness assessment process was part of the overall operational activities and covered by the existing technical assistance of WHO, UNICEF, and partners, no additional costs were budgeted for the activity. However, inclusion of more districts, blocks, and health centers in the process could help to ensure homogeneous quality of SIA implementation.

The findings in this report are subject to at least two limitations. First, the selection of areas for readiness assessments included in this report was purposeful, and no control groups were available for comparison. Second, the impact of the readiness assessments on achieving the ≥95% SIA coverage target was not assessed by post-SIA surveys because of time and resource limitations and lack of a comparison group.

The WHO South-East Asia Region aims to vaccinate >500 million children with measles-rubella vaccine through SIAs by 2019. The experience with pre-SIA assessments in India reported here will help improve preparedness for high-quality SIAs, ensuring high vaccination coverage to achieve the regional goal of measles elimination and rubella and congenital rubella syndrome control by 2020.

**Summary**

**What is already known about this topic?**

India has adopted a goal for measles elimination and rubella and congenital rubella syndrome control by 2020 by achieving high coverage with 2 routine doses of measles-containing vaccine and supplemental immunization activities (SIAs), which require substantial preparation.

**What is added by this report?**

Two pre-SIA readiness assessments in 24 districts in three states provided feedback to decision-makers that led to corrective actions. Readiness improved from 33% to 79% between the two assessments.

**What are the implications for public health practice?**

The WHO South-East Asia Region aims to vaccinate >500 million children with measles-rubella vaccine through SIAs by 2019. The experience with pre-SIA assessments can help improve preparedness and ensure high coverage through SIAs in the region.

## Footnotes

* The WHO South-East Asia Region consists of 11 countries: Bangladesh, Bhutan, India, Indonesia, Maldives, Myanmar, Nepal, North Korea, Sri Lanka, Thailand, and Timor-Leste.

† Measles elimination is defined as the absence of endemic measles cases for a period of ≥12 months, in the presence of adequate surveillance. One indicator of measles elimination is a sustained measles incidence of less than one case per 1 million population. Rubella/congenital rubella syndrome control is defined as ≥95% reduction in disease prevalence from 2013 levels.

§ India states and union territories and target populations (in millions) included in SIA phase 1 were Tamil Nadu (17.6), Karnataka (16.03), Goa (0.32), Puducherry (0.30), Lakshadweep (0.16), and in SIA phase 2 were Andhra Pradesh (11.85), Chandigarh (0.31), Daman & Diu (0.05), Dadra & Nagar Haveli (0.11), Telangana (9.00), Himachal Pradesh (1.77), Uttarakhand (2.80), and Kerala (7.65).

¶ SIAs generally are carried out using two target age ranges. An initial, nationwide catch-up SIA focuses on all children aged 9 months–14 years, with the goal of eliminating susceptibility to measles in the general population. Periodic follow-up SIAs then focus on all children born since the last SIA. Follow-up SIAs generally are conducted nationwide every 2–4 years and focus on children aged 9–59 months; their goal is to eliminate any measles susceptibility that has developed in recent birth cohorts and to protect children who did not respond to the routine first dose of measles-containing vaccine.

†† A block is the third administrative unit, found within a district, and a planning unit is the lowest administrative unit of the health system, found within a block.
